# New surgery technique for refractory macular hole guided by intraoperative OCT: free internal limiting membrane flap and autologous blood clot

**DOI:** 10.1186/s40942-025-00681-6

**Published:** 2025-05-27

**Authors:** Thais Marino de Azeredo Bastos, Laís Lauria Neves, Letícia Pinheiro de Freitas, David Leonardo Cruvinel Isaac, Antônio Marcelo Barbante Casella, Marcos Pereira Ávila

**Affiliations:** 1 Ophthalmology Reference Center at the Federal University of Goiás (CEROF-UFG), Goiânia, GO 74605-020 Brazil; 2 Centro Brasileiro de Cirurgia de Olhos (CBCO), Av. T-2, 401 – St. Bueno, Goiânia, GO 74210-010 Brazil; 3 Departament of Ophthalmology, Estadual de Londrina, UEL, Londrina, PR Brazil; 4 Division of Ophthalmology, Ribeirão Preto Medical School, University of São Paulo, São Paulo Ribeirão Preto, Brazil

**Keywords:** i-OCT, Refractory macular hole, Vitrectomy, ILM peeling, Blood clot

## Abstract

**Background:**

Present a new surgical technique which involves using pars plana vitrectomy (PPV) combined with a free internal limiting membrane (ILM) flap anchored by an intraoperatively obtained blood clot, assisted by intraoperative optical coherence tomography (i-OCT).

**Methods:**

This is a prospective interventional non-comparative study with three cases of recurrent or refractory macular hole treated with a new surgical technique using autologous blood clot obtained from the retinal vessels.

**Results:**

This study included 3 eyes of 3 patients. All patients were previously treated with a PPV and internal limiting membrane peeling. One week post-operatively, all operated eyes showed closure of the macular hole. No complications secondary to the surgical technique occurred.

**Conclusion:**

This is a small case series that obtained anatomical success in the macular hole closure with a reproducible technique involving the use of i-OCT to position a free ILM flap, anchored by a blood clot obtained from per operatory retinal bleeding. Further controlled research needs to be done to prove the efficacy.

## Background

A macular hole (MH) is defined as a full-thickness defect in the neurosensory retina involving the fovea. It can be caused by various etiologies [1], including high myopia and trauma; however, most cases are classified as idiopathic macular hole (IMH), for which no specific cause is identified. IMH is typically caused by vitreous traction on the fovea, occurring in both anteroposterior and tangential directions [2–4]. IMH is generally a unilateral condition seen predominantly in middle-aged and older women [5].

Spontaneous closure of MH is rare, occurring in approximately 3–15% of cases [6], particularly in the absence of vitreomacular adhesion. In most cases, surgical intervention with pars plana vitrectomy (PPV) is required. This procedure involves releasing vitreous adhesion or traction on the retina, followed by internal limiting membrane (ILM) peeling and gas tamponade, which enhances both visual and anatomical outcomes [7]. The standard protocol for this technique also involves postoperative face-down positioning. This approach has demonstrated a high success rate, with over 90% of cases achieving macular hole closure, making it the current standard of care.

Recent challenges, however, have focused on identifying optimal surgical approaches for refractory, recurrent, and complex macular holes, including those with a large diameter (>600 μm) or those associated with high myopia, where the success rate using conventional techniques is significantly lower [8]. Most recent reports indicate that the anatomical closure rate after vitrectomy with ILM peeling surgery declines from 80 to 56% for large macular holes [9].

Many surgical modifications have been proposed to address these complex cases, including the inverted ILM flap, free ILM flap, amniotic membrane transplantation, hydrodissection, and relaxing retinotomy, among others. These techniques, however, have limitations, such as insufficient tissue for an inverted flap, loss of the free flap, and difficulty in obtaining amniotic membrane. Additionally, the use of autologous blood, either alone or combined with an ILM flap, has been reported in a few cases in the literature [1].

The objective of this report is to describe three successful cases of refractory MH that were initially treated with the standard surgical protocol but required reoperation in which a free ILM flap anchored by an intraoperatively obtained autologous blood clot was used. Since there is no consensus on the ideal technique for the treatment of complex macular holes, such as refractory or recurrent holes, the technique described in this article may serve as an additional therapeutic option, especially in cases where a free flap is intentionally or inadvertently created. Intraoperative optical coherence tomography (i-OCT) was used to assist in the identification and precise positioning of the ILM flap.

## Materials and methods

This is a prospective interventional non-comparative study with 3 cases of refractory macular hole, undergoing vitreoretinal surgery at the Brazilian Eye Surgery Center (CBCO) by a single surgeon, one of the authors of this study (MPA), in Goiânia, Goiás, Brazil. All of them were previously treated with PPV and ILM peeling but failed to achieve macular hole closure. We treated such cases with the PPV, ILM peeling, creation of a free flap and its placement anchored in a drop of blood inside the macular hole, finished with a fluid-air exchange, which was subsequently replaced with 13% C3F8 gas (Ispan, Alcon Laboratories, Fort Woth, TX, USA). Its correct positioning was confirmed via intraoperative optical coherence tomography (iOCT) (Artevo 800, Carl Zeiss Meditec, Jena, Germany).

The data collected before the surgery was: best-corrected Snellen visual acuity, intraocular pressure, fundus photograph, and spectral-domain optical coherence tomography (SPECTRALIS^®^, Heidelberg Engineering, Germany). The diameter of the MH was measured on OCT.

This study adhered to the tenets of the Declaration of Helsinki and was approved by the institutional review boards of the participating institutions. All subjects voluntarily agreed to participate by signing the informed consent form.

## Results

This study included 3 eyes of 3 patients. Two patients were female, and one was male. All of them were previously treated with a PPV and internal limiting membrane peeling. The interval between the first and second surgery was 4 months in the first case, 6 months in the second case and 8 months in the third. The preoperatively best corrected visual acuity 20/200 in two patients and 20/60 in one patient. Post-operatively, all operated eyes showed complete closure of the macular hole. No complications secondary to the surgical technique occurred. There was a limitation since one patient (case 3) lost the follow up. The demographic data and results are summarized in Table 1.

**Table 1:** Summary of all three cases

	Case 1	Case 2	Case 3
Age	75 years old	56 years old	65 years old
Gender	Female	Male	Female
Macular hole size (µm)	640	391	529
Interval between first and second surgery (months)	4	4	8
BCVA pre op	20/200	20/200	20/60
Surgery technique	PPV + free flap + autologous blood + fluid air exchange + 13% C3F8 tamponade	PPV + free flap + autologous blood + fluid air exchange + 13% C3F8 tamponade	PPV + free flap + autologous blood + fluid air exchange + silicon oil
Tamponade	13% C3F8	13% C3F8	Silicon oil
Use of iOCT	Yes	Yes	Yes
Hole closure after second surgery	Yes	Yes	Yes

Best corrected visual acuity (BCVA), pars plana vitrectomy (PPV), perfluoropropane (C3F8)

### Surgical technique

The patients had a 25-gauge PPV (Constellation Vision System, Alcon Labs, Fort Worth, TX, United States of America), with staining of the macula with brilliant blue 0.05% (Opht-Blue, Ophthalmos, Sao Paulo, Brazil). A free flap far from the macular hole was created with a 25-gauge ILM forceps (Finesse Sharkskin ILM Forceps, Alcon Labs, Fort Worth, TX, USA). The flap was delicately positioned inside the macular hole anchored on a drop of autologous blood. This blood was obtained from the venous retinal capillary circulation through a delicate maneuver with the Finesse Sharkskin ILM Forceps^®^ (Alcon), under the surgeon’s control. After this maneuver, the ILM flap became indistinguishable as it merged with the blood clot; however, its correct positioning was confirmed via intraoperative optical coherence tomography (iOCT) (Artevo 800, Carl Zeiss Meditec, Jena, Germany). At the end of the surgery, a fluid-air exchange was performed, which was subsequently replaced with 13% C3F8 gas (Ispan, Alcon Laboratories, Fort Woth, TX, USA) or silicone oil (Oxane 5700, Bausch & Lomb, USA). The patients were instructed to maintain a face-down position for 18 h per day for one week.

### Case presentation

#### Case no. 1

A 75-year-old female patient presented to our institution with a history of prior pars plana vitrectomy (PPV) and internal limiting membrane (ILM) peeling in both eyes for the treatment of macular holes (MH).

At the initial examination, her best-corrected visual acuity (BCVA) was 20/200 in the right eye (OD) and 20/30 in the left eye (OS). Intraocular pressure was 12 mmHg in the OD and 10 mmHg in the OS. Slit-lamp biomicroscopy revealed a well-positioned intraocular lens in both eyes.

**Fig. 1:**
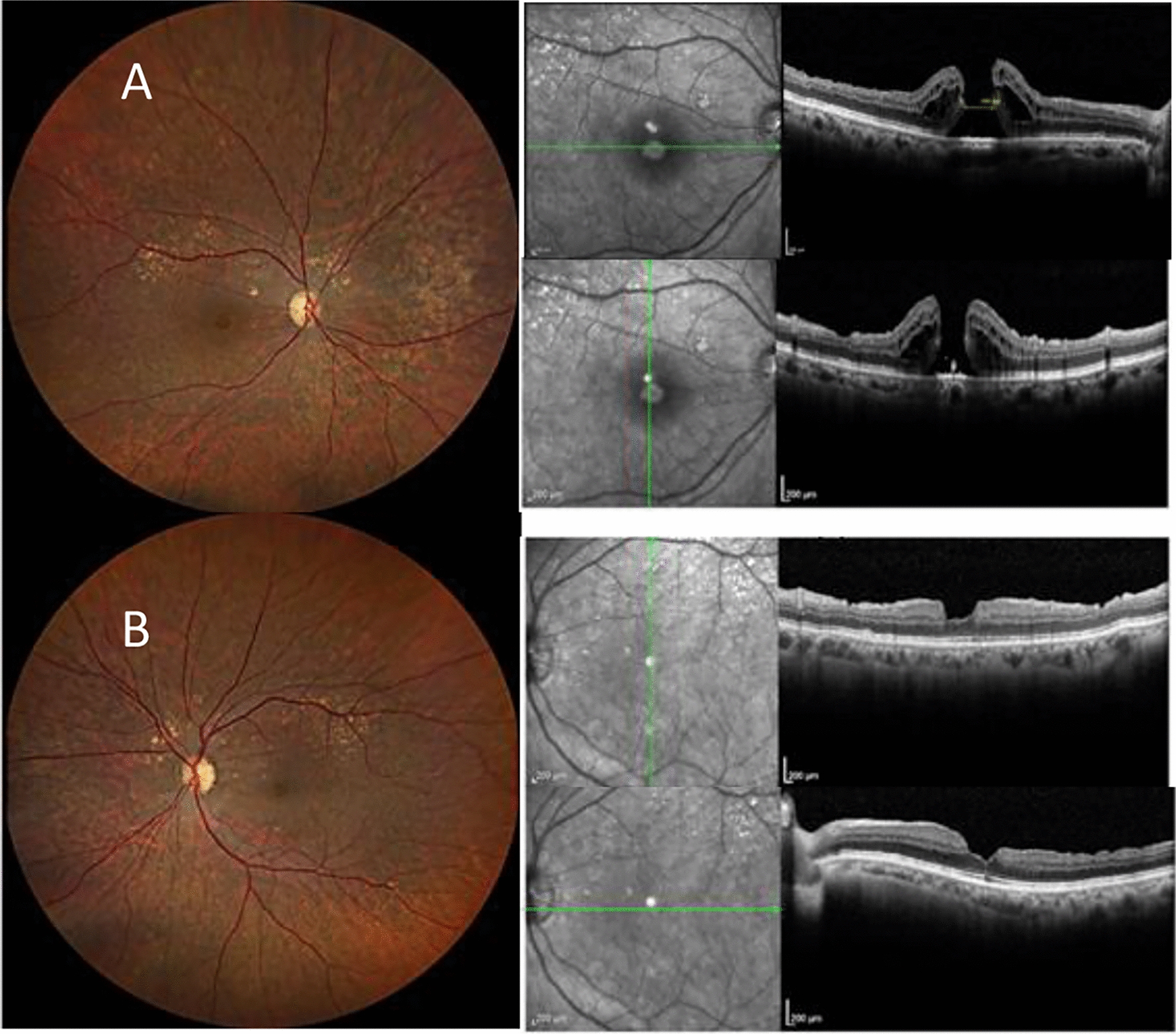
**A** Retinography and OCT of the right eye, demonstrating a full-thickness macular hole with intraretinal cysts surrounding the foveal area after previous pars plana vitrectomy (PPV) for macular hole repair. The infrared image adjacent to the OCT highlights the boundaries of the previously peeled internal limiting membrane (ILM) in the macular region. **B** Retinography and OCT of the fellow eye (same patient as in **A**), showing a successfully closed macular hole after PPV with irregularities in the vitreoretinal interface

Optical coherence tomography (OCT) demonstrated a refractory full-thickness macular hole with a diameter of 640 μm was observed in OD (Fig. 1A) and successful closure of the macular hole in the OS (Fig. 1B).

### Surgical technique

**Fig. 2:**
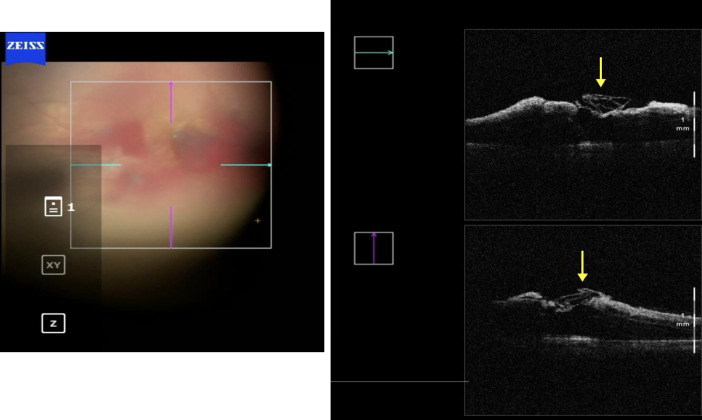
Intraoperative OCT image demonstrating the anchoring of the free ILM flap within the macular hole by a whole blood clot. The correct positioning of the flap was confirmed intraoperatively using i-OCT

During the PPV of the right eye (Constellation Vision System, Alcon Labs, Fort Worth, TX, United States of America), the posterior hyaloid was found to be detached. After staining with brilliant blue 0.05% (Opht-Blue, Ophthalmos, Sao Paulo, Brazil), the boundaries of the previously peeled ILM around the macular hole were clearly visible, and a flap was created temporal superior to the macula area with a 25-gauge ILM forceps (Finesse Sharkskin ILM Forceps, Alcon Labs, Fort Worth, TX, USA). The initial plan was to perform a pediculated ILM flap; however, due to extensive peeling during the first surgery, the everted flap obtained was insufficient to cover the macular hole. Consequently, we converted the dissected ILM into a free flap. To anchor the flap, a drop of blood was obtained from the venous retinal capillary circulation through a delicate maneuver with the Finesse Sharkskin ILM Forceps^®^ (Alcon), under the surgeon’s control. Using ILM forceps, the free ILM flap, which was initially attached to the vitreoretinal interface, was mobilized and positioned over the macular hole. It was then anchored into the hole edges using the drop of blood. After this maneuver, the ILM flap became indistinguishable as it merged with the blood clot; however, its correct positioning was confirmed via intraoperative optical coherence tomography (iOCT) (Artevo 800, Carl Zeiss Meditec, Jena, Germany) (Fig. 2). At the end of the surgery, a fluid-air exchange was performed, which was subsequently replaced with 13% C3F8 gas (Ispan, Alcon Laboratories, Fort Woth, TX, USA). The patient was instructed to maintain a face-down position for 18 h per day for one week.

### Follow-up

**Fig. 3:**
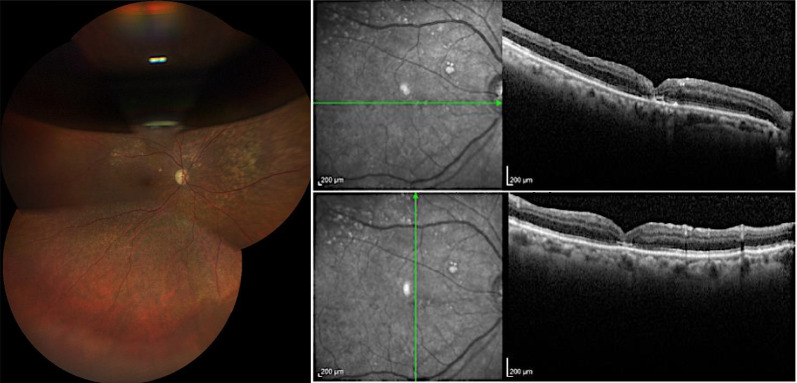
One month post-operative retinography and OCT of the right eye demonstrating complete macular hole closure

At one-month postoperative follow-up, optical coherence tomography (OCT) revealed complete closure of the macular hole, with remnants of the internal limiting membrane (ILM) visible within the treated area. Irregularities in the outer retinal layers were observed, along with a hyperreflective area in the ellipsoid zone at the fovea (Fig. 3). The best-corrected visual acuity (BCVA) in the right eye (OD) had improved to 20/50.

**Fig. 4:**
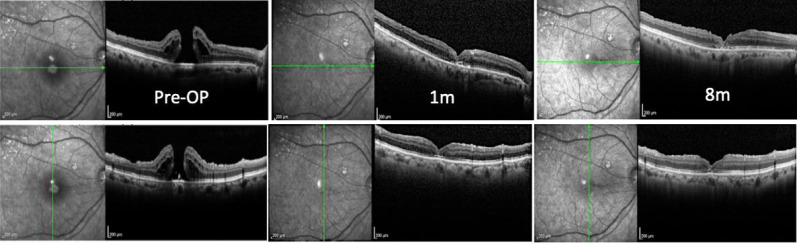
OCT of the right eye at 8-month follow-up after macular hole surgery

The patient was followed for an additional 8 months, during which the condition remained stable (Fig. 4).

#### Case no. 2

A 56-year-old male patient presented to our hospital with complaints of progressively worsening vision in his right eye (OD).

At the initial examination, his best-corrected visual acuity (BCVA) was 20/200 in the right eye (OD) and 20/20 in the left eye (OS). The patient was phakic, with no significant cataract.

**Fig. 5:**
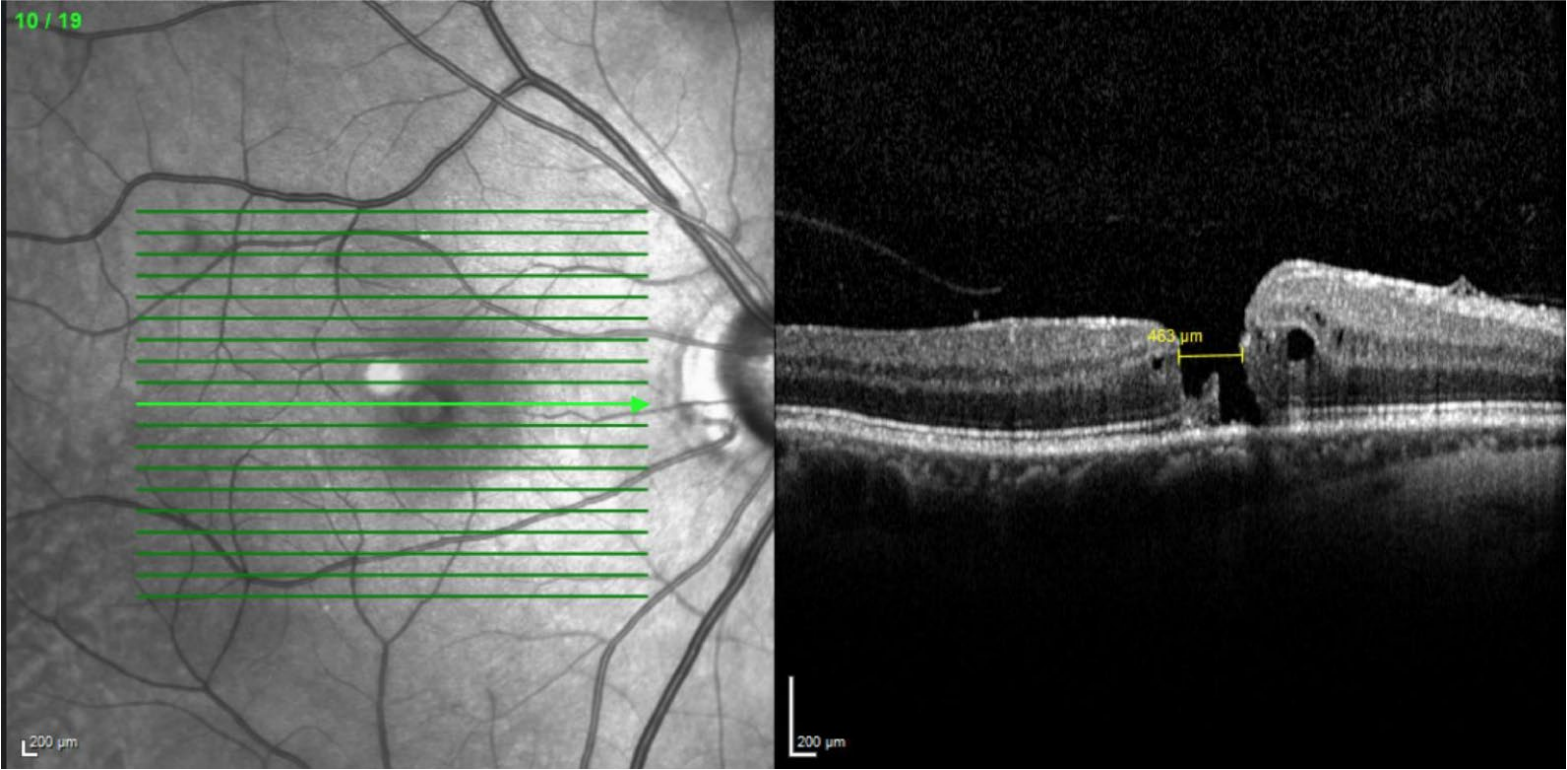
OCT image showing a full-thickness macular hole in the right eye (OD)

In the right eye, a full-thickness macular hole with a diameter of 463 μm was observed (Fig. 5). Optical coherence tomography (OCT) of the left eye revealed no abnormalities.

### Surgical technique

A pars plana vitrectomy (PPV) was performed (Constellation Vision System, Alcon Labs, Fort Worth, TX, United States of America). Following the detachment of the posterior hyaloid, brilliant blue dye 0.05% (Opht-Blue, Ophthalmos, Sao Paulo, Brazil)was injected into the vitreous cavity, and an extensive internal limiting membrane (ILM) peeling was performed using a 25-gauge ILM forceps (Finesse Sharkskin ILM Forceps, Alcon Labs, Fort Worth, TX, USA) around the macular hole. Intraoperative optical coherence tomography (i-OCT) (Artevo 800, Carl Zeiss Meditec, Jena, Germany) was used during the procedure, and a thicker membrane adjacent to the hole edges, presumed to be an epiretinal proliferation, was identified and peeled. A fluid-air exchange was subsequently performed, followed by the injection of 13% C3F8 gas (Ispan, Alcon Laboratories, Fort Woth, TX, USA). The patient was advised to maintain a face-down position postoperatively.

**Fig. 6:**
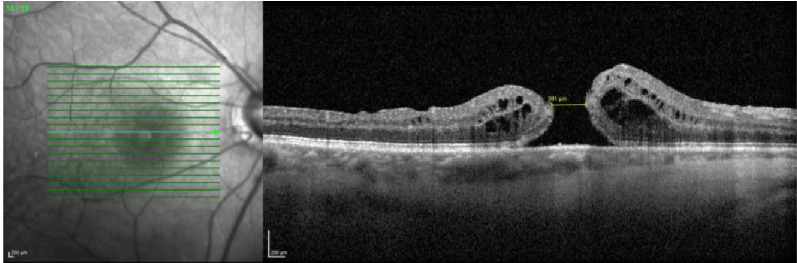
OCT image following PPV with ILM peeling, showing a refractory macular hole

**Fig. 7:**
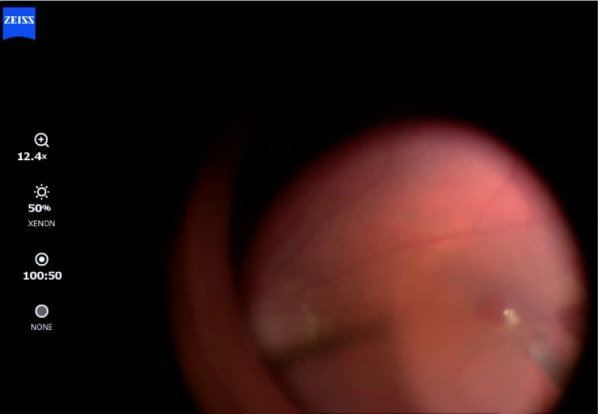
Intraoperative image capturing the moment of ILM flap placement within the macular hole, which already contained a blood clot at its base

**Fig. 8:**
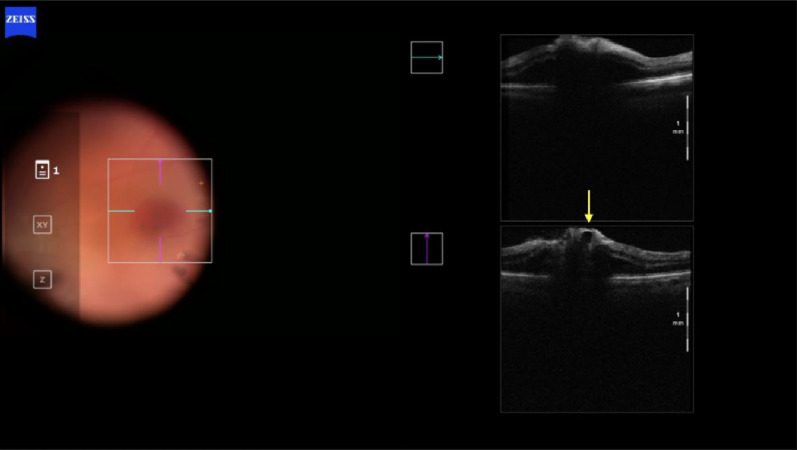
Intraoperative OCT image showing the mixture of the ILM flap and blood clot within the macular hole

At the six-month follow-up, closure of the macular hole had not occurred (Fig. 6), and the hole’s diameter had decreased to 391 μm. A decision was made to re-treat the refractory macular hole. Similar to the previous case, due to the extensive ILM peeling performed during the initial surgery, a pediculated ILM flap was not feasible. Therefore, a free flap technique was chosen. To anchor the flap, a drop blood was obtained from the venous retinal capillary circulation as previously described. The ILM free flap was created and positioned over the macular hole, anchoring it with the blood clot (Fig. 7). The proper positioning of the flap was confirmed via i-OCT (Fig. 8).

At the end of the surgery, a fluid-air exchange was performed, followed by the injection of 13% C3F8 gas. The patient was instructed to maintain a face-down position for one week.

### Follow-up

**Fig. 9:**
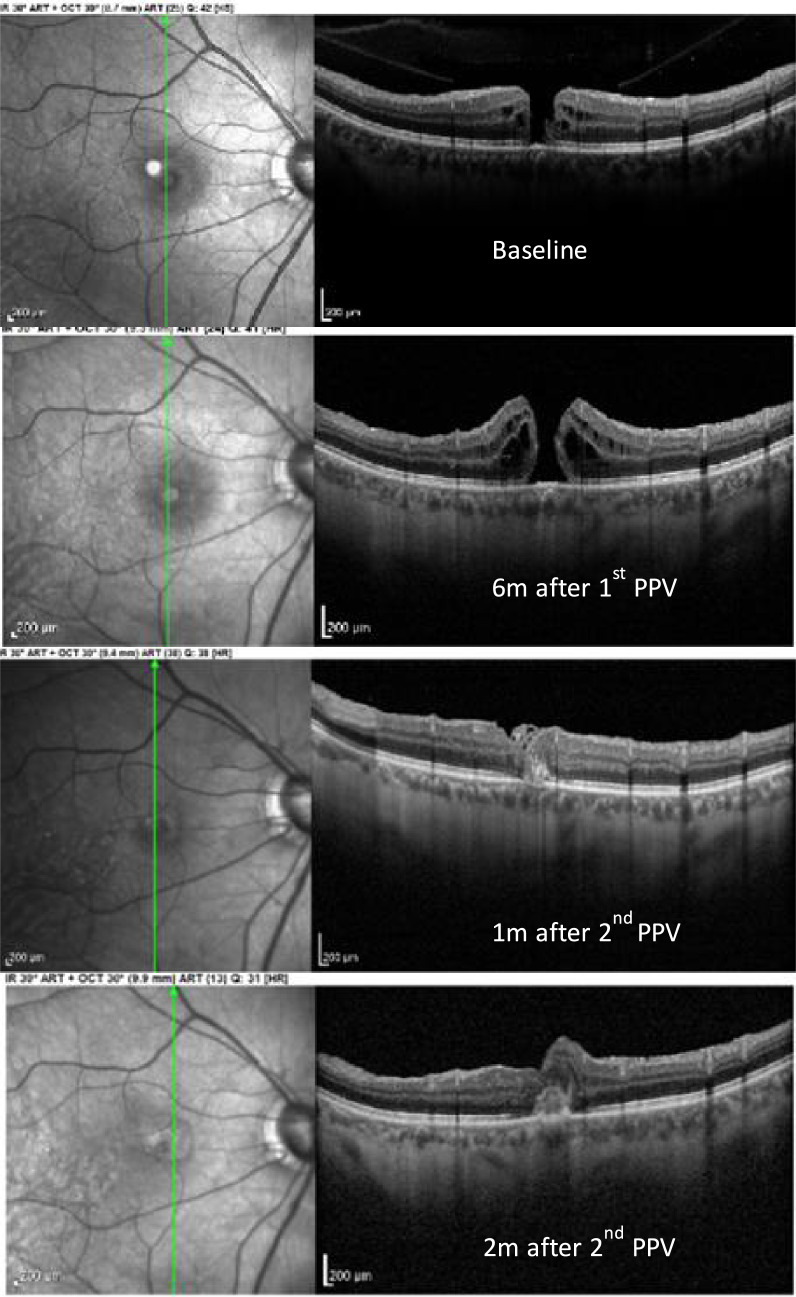
Comparative OCT images illustrating the progression of the macular hole at baseline, the refractory macular hole after the initial surgery, and the follow-up at 1 and 2 months post-second surgery

At the one-month postoperative follow-up, OCT revealed complete closure of the macular hole, with remnants of the ILM flap and hyperreflective material in the outer retinal layers. Two months after the final surgery, the ILM flap was fully incorporated, and the hyperreflective material persisted (Fig. 9). The best-corrected visual acuity (BCVA) in the right eye (OD) was 20/150.

#### Case no. 3

A 65-year-old female patient, previously treated with cataract surgery, presented to our hospital with complaints of absence of improvement in vision in her right eye (OD) after surgery.

At the initial examination, her best-corrected visual acuity (BCVA) was 20/60 in the right eye (OD) and 20/20 in the left eye (OS).

**Fig. 10:**
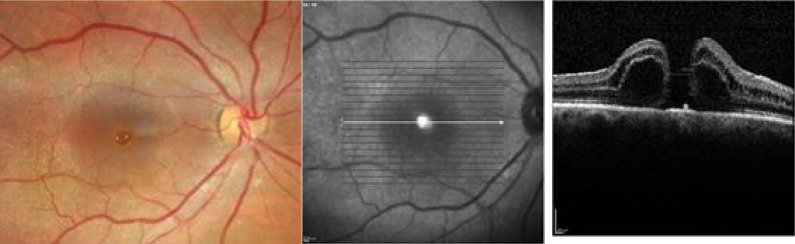
Retinography and OCT of the right eye, demonstrating a full-thickness macular hole with intraretinal cysts surrounding the fovea

In the right eye, a full-thickness macular hole with a diameter of 529 μm was observed (Fig. 10). Optical coherence tomography (OCT) of the left eye revealed no abnormalities.

### Surgical technique

A pars plana vitrectomy (PPV) was performed (Constellation Vision System, Alcon Labs, Fort Worth, TX, United States of America). Following the detachment of the posterior hyaloid, brilliant blue dye 0.05% (Opht-Blue, Ophthalmos, Sao Paulo, Brazil)was injected into the vitreous cavity, and an extensive internal limiting membrane (ILM) peeling was performed using a 25-gauge ILM forceps (Finesse Sharkskin ILM Forceps, Alcon Labs, Fort Worth, TX, USA) around the macular hole. Intraoperative optical coherence tomography (i-OCT) (Artevo 800, Carl Zeiss Meditec, Jena, Germany) was used during the procedure, and a thicker membrane adjacent to the hole edges, presumed to be an epiretinal proliferation, was identified and peeled. A fluid-air exchange was subsequently performed, followed by the injection of 10% SF6 gas (Ispan, Alcon Laboratories, Fort Woth, TX, USA). The patient was advised to maintain a face-down position postoperatively.

**Fig. 11:**
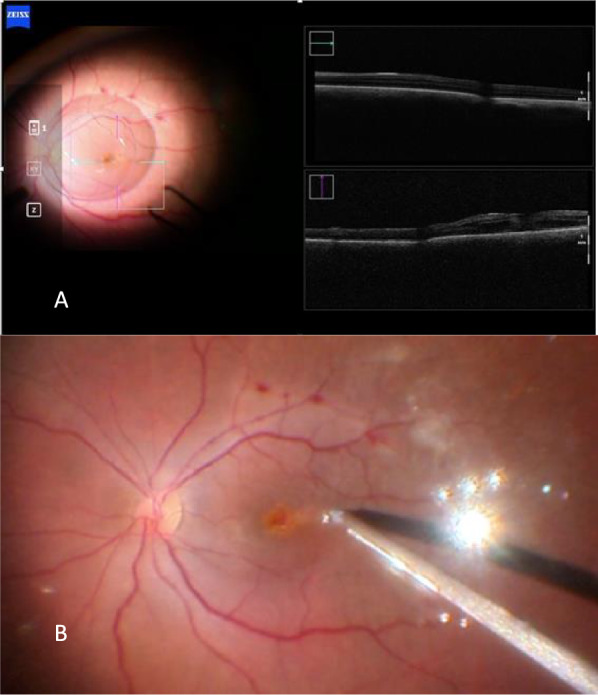
Intra-operative images. **A** A intraoperative OCT was used to confirm The ILM free flap inside the macular hole. **B** Image after fluid-air exchange, showing the macular hole with a drop of blood

At the eight-month follow-up, the macular hole was still open. We decided to re-treat the refractory macular hole. Like the previous cases, a free flap technique was chosen. We used perfluorcarbon to maintain the position of the flap. During the fluid-air exchange, a jet stream occurred. Because of that, the flap was dislocated from its proper position. A drop of blood was obtained to anchor the flap. The proper positioning of the flap was confirmed via i-OCT (Fig. 11).

At the end of the surgery, a fluid-air exchange was performed. Then, injection of silicone oil (Oxane 5700, Bausch & Lomb, USA) was performed, as the patient had to travel by plane in the following days, we couldn’t use gas to tamponade because of the risk of expansion. The patient was instructed to maintain a face-down position for one week.

### Follow-up

**Fig. 12:**
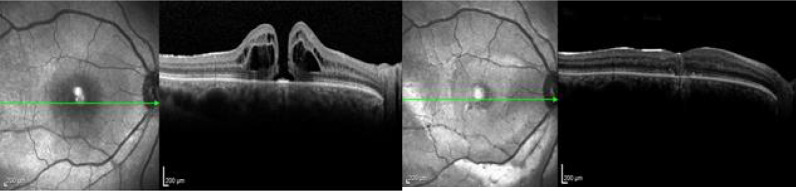
OCT image of before and two days after surgery, showing complete closure of the macular hole

**Fig. 13:**
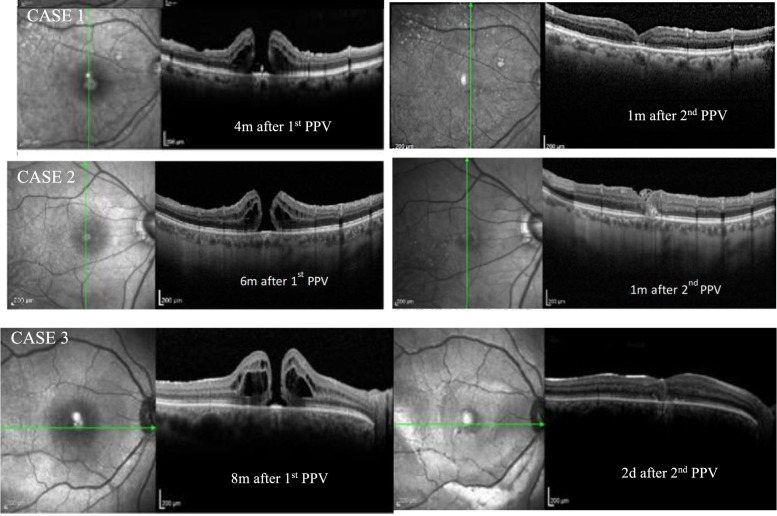
OCT image of all cases before and after surgery, showing complete closure of the macular hole

After two days, in the postoperative follow-up, OCT revealed complete closure of the macular hole (Fig. 12). Unfortunately, the patient lost follow-up after that, what confine our knowledge of the long-term result. The OCTs before and after surgery of all three cases are summarized in Fig. 13.

## Discussion

Idiopathic macular hole is a significant vision-threatening condition, particularly affecting the middle-aged population. The success of surgical intervention is influenced by several factors, including macular hole diameter, the presence of an epiretinal membrane (ERM), postoperative positioning, and the occurrence of cystoid macular edema (CME). Given these variables, it is essential for surgeons to master a wide range of surgical techniques to effectively manage complex macular holes [10].

Pars plana vitrectomy (PPV) for macular hole repair was first introduced by Kelly in 1991 [11]. The procedure involved performing a vitrectomy, inducing posterior vitreous detachment, peeling any fibrous membranes adherent to the retina, filling the eye with a long-acting gas tamponade, and requiring strict face-down positioning in the postoperative period. Subsequent evidence demonstrated that peeling the internal limiting membrane (ILM) improved macular hole closure rates, and this step was incorporated into the surgical routine by many surgeons [12]. More recently, the inverted flap technique, described by Michalewska et al. [13], has been widely adopted and further modified by various surgeons. These techniques have contributed to the understanding that placing the ILM over the macular hole promotes closure, leading to both anatomical and functional success.

In 2015, Lai et al. introduced the concept of combining an inverted ILM flap with autologous blood, which was obtained from the patient’s antecubital vein and injected into the vitreous cavity [14]. This technique achieved macular hole closure in 96% of cases. More recently, Hu et al. compared the inverted ILM flap with autologous blood (also obtained from the patient’s antecubital vein) to ILM insertion alone for the repair of refractory macular holes in 52 eyes, demonstrating superior outcomes with the former technique [15]. However, to date, there are no reported cases in the literature where a blood clot derived directly from retinal vascularization was used as in this report.

One potential advantage of using retinal blood is that it eliminates the need to draw autologous blood from another site, potentially reducing the risk of contamination. In cases where bleeding occurs during PPV, this retinal blood can be utilized for the procedure.

In previous studies where a free ILM flap was used, a pediculated flap was first positioned inside the macular hole, subsequently, a drop of blood was applied to seal the hole and then the remaining of the ILM was peeled [14, 15]. It is well known that positioning the flap within the macular hole while the vitreous cavity is filled with balanced salt solution (BSS) can be technically challenging, with a high risk of flap loss. For this reason, some surgeons utilize perfluorocarbon (PFC) to assist with flap positioning, but this also carries a risk of losing the flap during the PFC-air exchange. In our approach, we propose placing a drop of blood in the macular hole before positioning the ILM flap, using the clot as an anchor to secure the flap and reduce the risk of displacement into the vitreous cavity.

Since intraoperative OCT (i-OCT) can be performed under air, this evaluation should be carried out both during the ILM peeling and flap positioning maneuvers, as well as after the fluid-air exchange, to confirm proper flap placement.

The blood clot may offer several benefits, including sealing the macular hole, facilitating reposition of the hole edges, and providing a scaffold for glial cell migration, which could promote closure [16]. Furthermore, the platelets and growth factors present in the blood clot may encourage tissue regeneration [16]. Additionally, the contraction of the blood clot might lead to centripetal movement of the retina toward the foveal center, thereby promoting hole closure [16]. Theoretically, there is a risk of exacerbated gliosis and the presence of intraretinal fibrin associated with the use of blood. Excessive gliosis could lead to the formation of scar tissue, limiting tissue remodeling and, consequently, visual prognosis. However, in the cited studies, such effects were not reported.

Autologous blood has traditionally been used as an adhesive to secure a free neurosensory retinal flap under gas tamponade in eyes that have undergone multiple surgeries where ILM flaps are no longer viable [17].

In these cases, we propose applying this adhesive property to an ILM-only flap. It remains uncertain whether the closure of the macular hole in these cases can be attributed to the ILM flap, the blood clot, or a combination of both. Our study is limited by the small number of cases and the absence of histopathological data, which could help elucidate the mechanisms responsible for macular hole closure, the structural reorganization of the tissues, as well as the specific role of each element (blood and internal limiting membrane flap) in the therapeutic success and the potential induction of complications. Further studies are needed to elucidate this mechanism and optimize treatment outcomes. This technique, despite its limitations, offers a viable option for recurrent or refractory macular holes, particularly in cases where extensive peeling of the internal limiting membrane has already been performed, preventing the creation of a pediculated flap. As described, other alternatives in such cases include the use of heterologous tissues, such as amniotic membrane—which is often difficult to obtain—autologous retinal transplantation, which carries surgical risks and technical challenges, or the use of a free flap, whose proper positioning is challenging. The proposed technique is an adaptation of previously described methods, in which blood is typically obtained from the cubital vein. In our variation, blood is drawn from the retinal circulation itself, reducing the risk of infection, offering a short learning curve, and facilitating both the positioning and adhesion of the free flap—making it a viable alternative for the challenging treatment of complex macular holes.

## Data Availability

No datasets were generated or analysed during the current study.
